# Causes, Nature and Treatment of Palsey and Apoplexy

**Published:** 1851-01

**Authors:** 


### ARTICLE II.

*On the Causes, Nature and Treatment of Palsy and Apoplexy: of the Forms, Seats, Complications, and Morbid relations of Paralytic and Apoplectie Diseases* By JAMES COPLAND, M. D. F. R. S., Fellow of the Royal College of Physicians; Honorary member of the American Philosophical Society, and of the Royal Academy of Medicine, of Belgium, Fellow of the Royal Medical and Chirurgical Societies of London and Berlin; lately consulting Physician to Queen Charlotte's Lying-in Hospital; Consulting, and lately Senior Physician to the Royal Infirmary for Diseases of Children; formerly Senior Physician to the South London Dispensary, etc. etc. Philadelphia, Lea & Blanchard, 1850, pp., 326, 12 mo., (From the Publishers, and for sale by S. C. Griggs & Co.)

This excellent and elaborate monograph is from the pen of the eminent author of the Dictionary of Practical Medicine, in which a portion of the volume has already appeared. But excellent and elaborate as it is, the volume does not present to our notice much that is really new, concerning the nature and treatment of Palsy and Apoplexy. Indeed, our author refers more frequently to old than modern authorities; and lays more stress on discoveries and doctrines promulgated by himself thirty years ago, than upon anything he has latterly achieved. But as a clear and concise summary of the present state of our knowledge of the diseases treated of, abounding in references to a vast array of authorities, both ancient and modern, all comprised in a small compass, it is certainly worthy a place in every Physician's library.

We apprehend that the most interesting portion of the volume, is that which treats of the connexion and complication of Palsy and Apoplexy with other diseases. These connections, according to our author, are not only important, but various and perplexing, in many cases rendering a diagnasis extremely difficult. We find these diseases in many cases connected with, or related to Insanity, Epilepsy, Chorea, Neuralgid, Hysterics, and even Rheumatism, requiring the most consummate skill and discrimination, for their proper diagnosis and treatment. These complications Dr. Copeland appears to have investigated with much attention, and we regard the results of his labors as very valulable.

Whilst giving due credit to other authors, for their discoveries in this department of Medicine, our author takes occasion to remind us in very emphatic terms that he will insist on hav- credit for his own,—that the laurels he has plucked, he will wear, gainsay it who may. This is all well enough. There are plenty of Medical as well as literary pirates, and for our own part, although as tender-hearted as Uncle-Toby himself, we can see them strung up at the yard arm, with entire complacency. Some may think Dr. Copeland a little querulous on this topic; but if a man does not defend his own right, it is morally certain no one else will. M.

---

